# Supplementary figures and images for: MG53 is not a critical regulator of insulin signaling pathway in skeletal muscle
Source: PLoS One. 2021 Feb 10;16(2):e0245179. doi: 10.1371/journal.pone.0245179 (PMC7875368; doi:10.1371/journal.pone.0245179)

Fig 2B

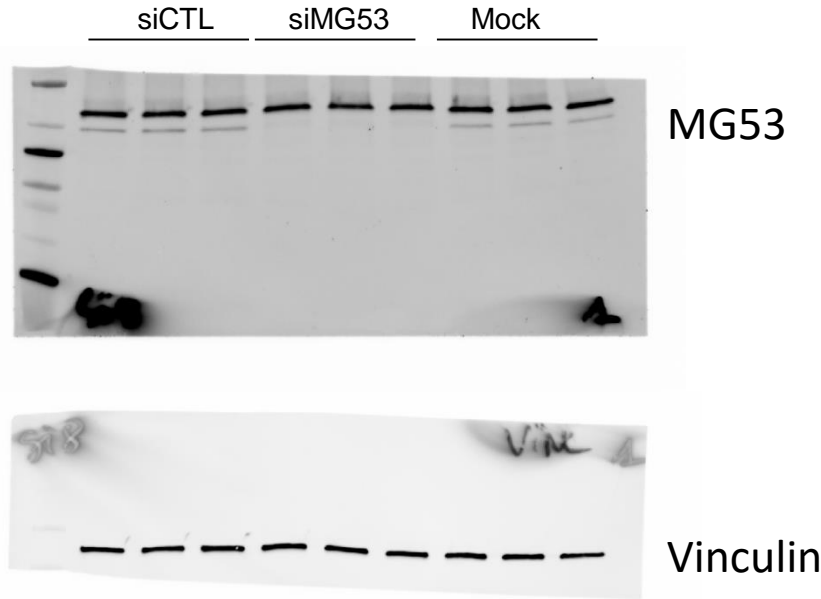

Fig 2C

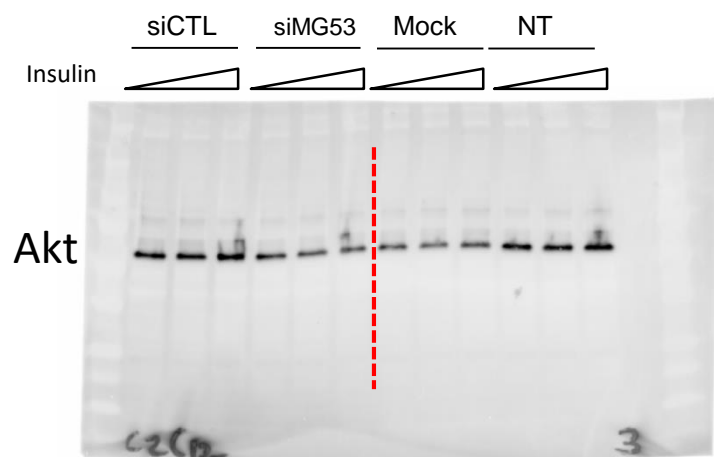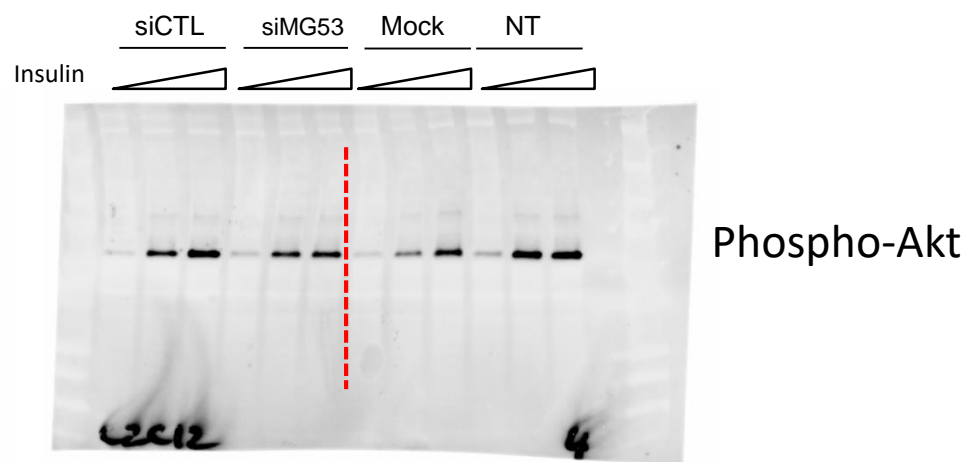

C2C12 (Fig 3A)

|                 | 1 h |   |   |   | 4 h |   |   |   |
|-----------------|-----|---|---|---|-----|---|---|---|
| hMG53 (30μg/ml) | -   | + | - | + | -   | + | - | + |
| Insulin         | -   | - | + | + | -   | - | + | + |

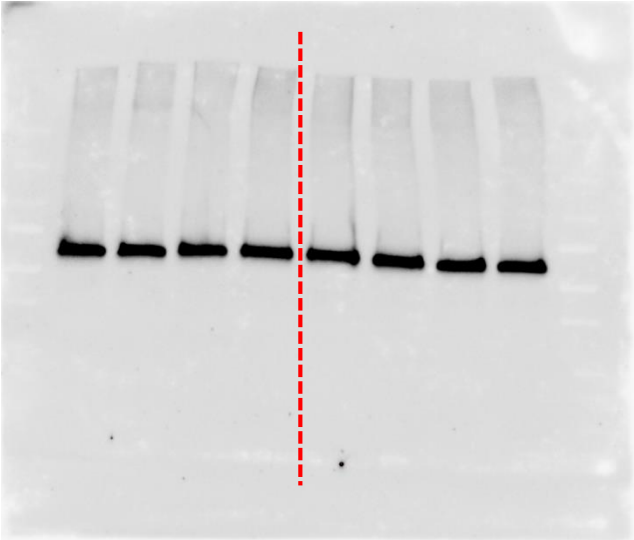

AKT

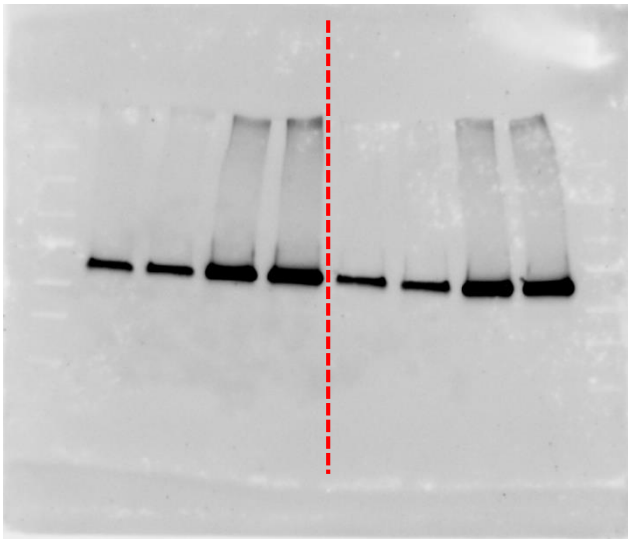

Phospho-AKT

HSMMs Fig 3B

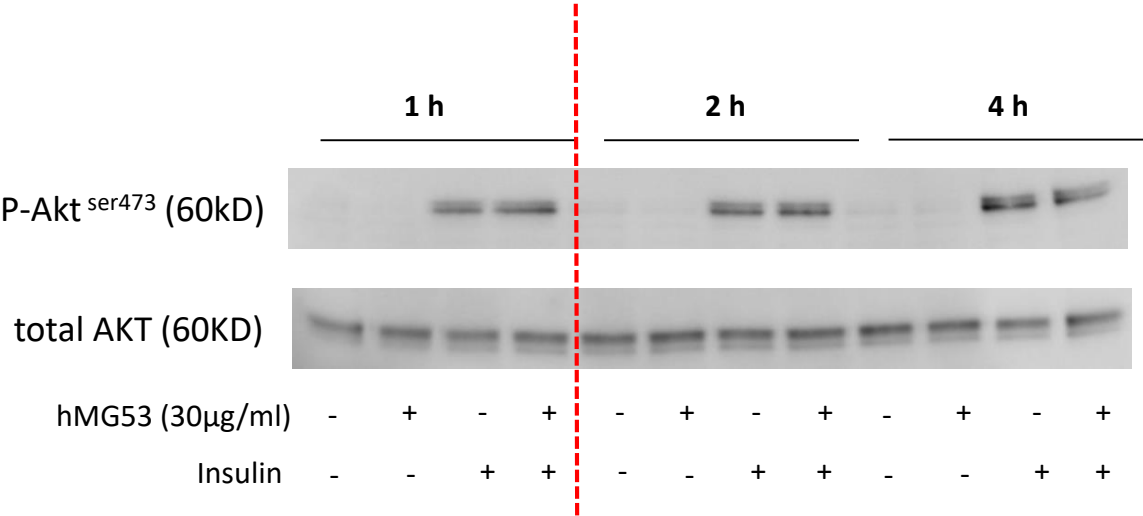

Fig4

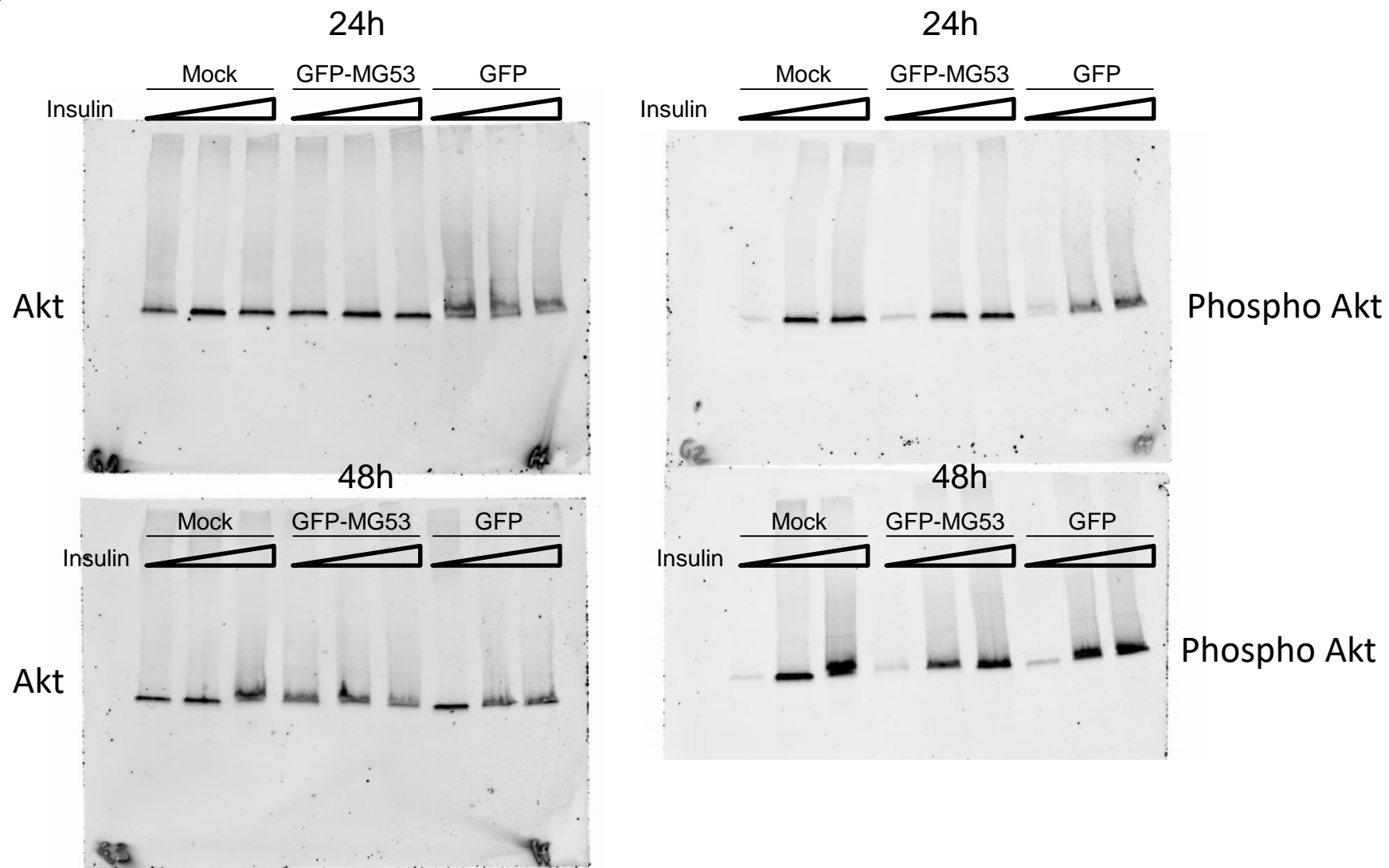

Fig4

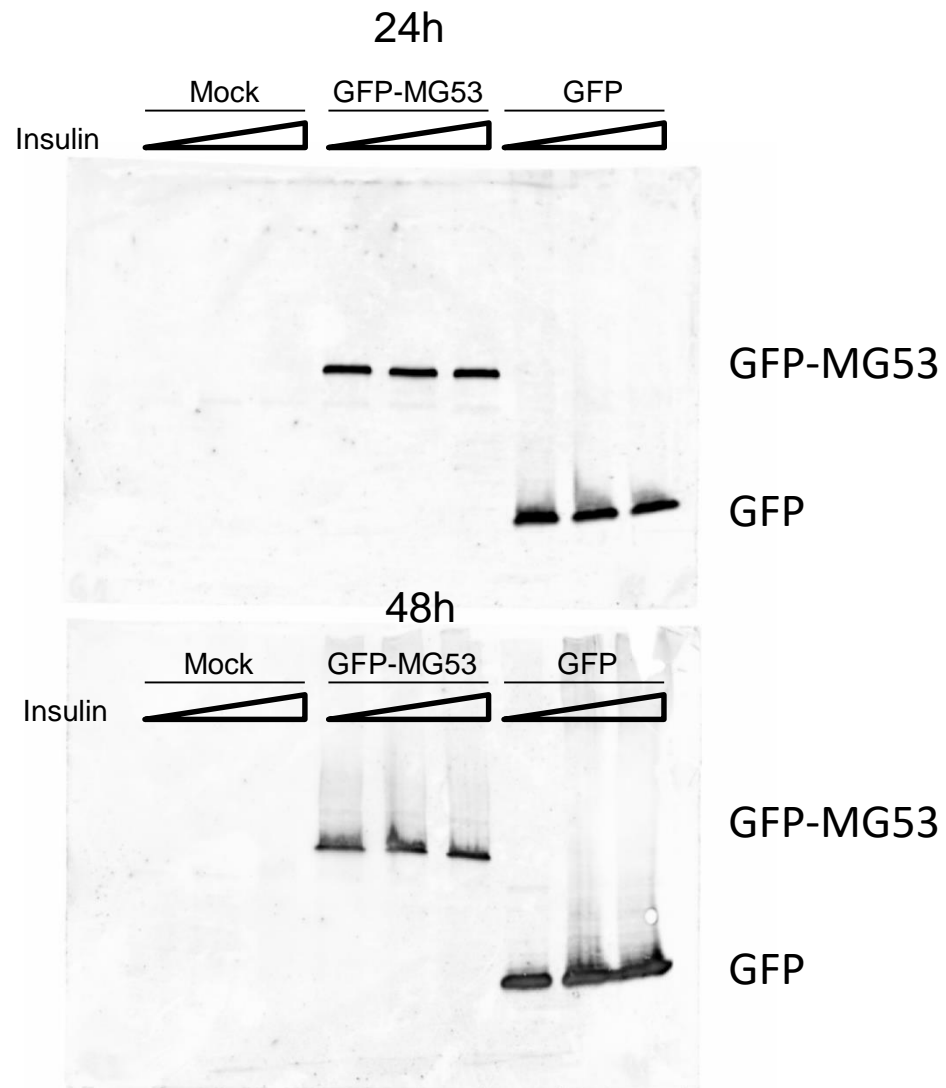

Fig5

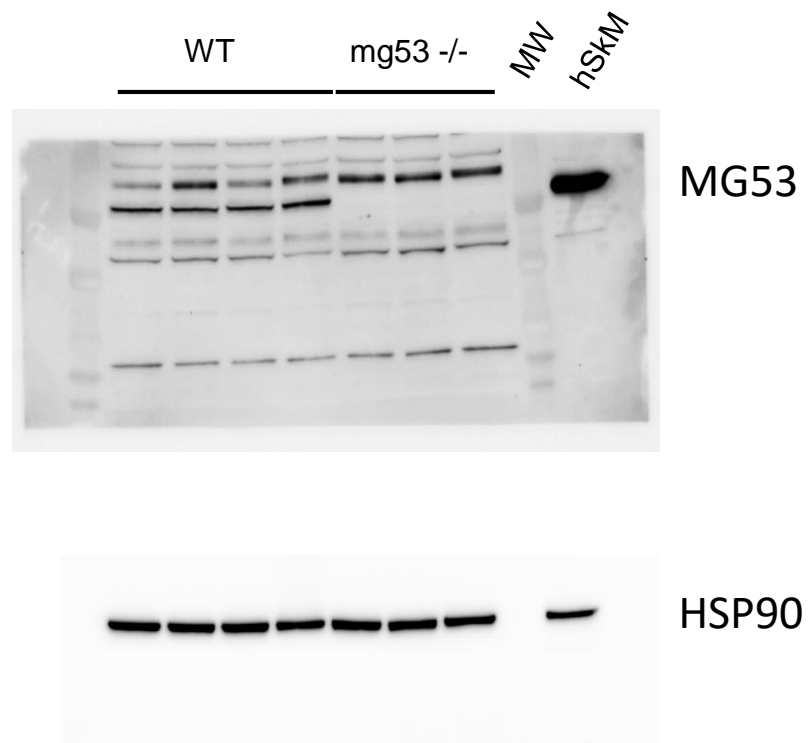

Supplement: S1 Raw images — (PDF) [file pone.0245179.s001.pdf]
